# Disorders of Bodily Identity and the Limits of Psychiatric Risk Prediction: A Case of Penile Self-Amputation

**DOI:** 10.1192/bjo.2026.11874

**Published:** 2026-06-30

**Authors:** Emeka Onochie, Saminathan Anand, Collins Esiwe, Sandeep Elluru

**Affiliations:** Lincolnshire Partnership NHS Foundation Trust, Lincoln, United Kingdom

## Abstract

**Aims:**

Body Integrity Dysphoria (BID) is a rare disorder characterised by a persistent sense of incongruence between bodily anatomy and experienced identity. ICD-11 situates BID within disorders of bodily experience, whereas DSM-5-TR lacks explicit diagnostic recognition, often leading to misclassification as body dysmorphic disorder, psychotic illness, or affective pathology. Extreme self-directed bodily modification remains exceptionally uncommon and poses profound diagnostic, ethical, and risk-assessment challenges.

**Methods:**

We describe a 71-year-old retired engineer with a lifelong pattern of non-psychotic, non-suicidal self-amputation. He had previously undergone medically indicated above-knee amputation due to osteomyelitis and deliberate mid-forearm self-amputation with an axe in midlife, preceded by longstanding discomfort with the limb and followed by subjective relief and euphoria. Approximately five years later, he developed persistent thoughts of genital amputation. These thoughts persisted for 15 years, were internally compelling but not delusional, and were unaccompanied by concerns regarding genital appearance or function, hallucinations, or depressive syndrome (PHQ-9=0; GAD-7=3). He repeatedly sought psychiatric and primary care support, expressing difficulty understanding his experiences and enquiring whether surgical intervention could be medically justified to minimise harm. He received antidepressant medication and psychological therapy.

Despite chronic ideation and a previous history of self-amputation, repeated clinical risk assessments concluded that imminent self-harm was unlikely, largely due to the absence of suicidality, affective instability, or behavioural escalation. Following his mother’s death, he executed a meticulously planned penile self-amputation after informing emergency services. Post-operatively, he reported relief and pride, without regret or depressive symptoms.

**Results:**

Phenomenologically, this presentation aligns more closely with BID than with body dysmorphic disorder or affective illness. Unlike body dysmorphic disorder, distress was not rooted in perceived defect or appearance but in a fundamental disturbance of bodily identity. Unlike suicidal self-harm, the act was purposeful, planned, and directed toward identity resolution rather than death. This case exposes limitations in contemporary risk frameworks, which prioritise acute, dynamic indicators of risk while undervaluing the clinical significance of chronic, identity-driven ideation. The persistence of symptoms was interpreted as stability rather than progressive consolidation of intent.

**Conclusion:**

This case underscores the need for phenomenologically informed, longitudinal approaches to psychiatric risk assessment and greater conceptual clarity regarding BID within diagnostic systems. Disorders of bodily identity can culminate in irreversible outcomes that evade conventional predictive models, highlighting the necessity of rethinking how risk, chronicity, and identity-related psychopathology are conceptualised in psychiatry.

